# Supplementary material for: Content-rich biological network constructed by mining PubMed abstracts
Source: BMC Bioinformatics. 2004 Oct 8;5:147. doi: 10.1186/1471-2105-5-147 (PMC528731; doi:10.1186/1471-2105-5-147)
Supplement: Additional File 2 — The original results of the above study (non-essential files are deleted to keep the file size under the limit set by BMC bioinformatics). [file 1471-2105-5-147-S2.bz2 › chilibotAdditionalFile2/dip05/226ID7982906E743/html/COPB_NEF.html]

 


 **COPB** and **NEF** 
  
Found 3 abstracts in PubMed, retrieved 3.  
 

 What does Google say? 
 PDF only 
| .edu only 

---

**Interactive relationship** (e.g. stimulation, inhibition, etc)

**Neutral relationship**- Physical interaction of the HIV 1  **Nef**  protein with beta COP  [ **COPB** ] , a component of non clathrin coated vesicles essential for membrane traffic.  Ref: 7982906 J Biol Chem, 1994
- **Nef**  induced CD4 degradation a diacidic based motif in  **Nef**  functions as a lysosomal targeting signal through the binding of beta COP  [ **COPB** ]  in endosomes.  Ref: 10199403 Cell, 1999
- These observations suggest that beta COP  [ **COPB** ]  might be one of the cellular mediators of  **Nef**  function in HIV 1 infected cells.  Ref: 7982906 J Biol Chem, 1994
- A sequence encompassing a critical acidic dipeptide, located nearby but distinct from the AP binding determinant of HIV 1  **Nef** , is responsible for beta COP  [ **COPB** ]  recruitment and for routing to lysosomes.  Ref: 10199403 Cell, 1999

**Non-interactive relationship** (e.g. studied together, co-existance, homology, etc.)

- Our results indicate that these acidic residues are not involved in the connection of  **Nef**  with the endocytic machinery through binding to beta COP  [ **COPB** ] .  Ref: 11264386 J Virol, 2001
- **Nef**  and beta COP  [ **COPB** ]  interacted in vitro and were found to be physically associated in HIV 1 infected cells by co immunoprecipitation.  Ref: 7982906 J Biol Chem, 1994
- Surprisingly, mutation of the diacidic sequence of  **Nef**  did not significantly affect its ability i to interact with beta COP  [ **COPB** ] , ii to downregulate CD4 cell surface expression, and iii to address an integral resident membrane protein containing  **Nef**  as the cytoplasmic domain to the endocytic pathway.  Ref: 11264386 J Virol, 2001
- **Nef**  induced CD4 downregulation a diacidic sequence in human immunodeficiency virus type 1  **Nef**  does not function as a protein sorting motif through direct binding to beta COP  [ **COPB** ] .  Ref: 11264386 J Virol, 2001
- Additional studies are thus required to characterize the residues of  **Nef**  involved in the binding to beta COP  [ **COPB** ]  and to evaluate the contribution of this interaction to the  **Nef**  induced perturbations of membrane trafficking.  Ref: 11264386 J Virol, 2001
